# Cytoreductive surgery and HIPEC in a 14 years old patient with peritoneal recurrence of adenocarcinoma of the right colon

**DOI:** 10.1016/j.ijscr.2019.02.046

**Published:** 2019-03-21

**Authors:** Lorena Sorrentino, Francesco Serra, Francesca Cabry, Stefano De Julis, Elisa Barbieri, Massimo Girardis, Pier Luca Ceccarelli, Roberta Gelmini

**Affiliations:** a Department of Surgery, University of Modena and Reggio Emilia – Policlinico of Modena, Via del Pozzo, 71, 41100 Modena, Italy; b Department of Anesthesiology, University of Modena and Reggio Emilia – Policlinico of Modena, Via del Pozzo, 71, 41100 Modena, Italy

**Keywords:** Cytoreductive surgery, Hyperthermic intraperitoneal chemotherapy (HIPEC), Pediatric surgery, Peritoneal surface malignancies, Oncological surgery

## Abstract

**Introduction:**

Cytoreductive surgery (CRS) and hyperthermic intraperitoneal chemotherapy (HIPEC) is nowadays a feasible and effective treatment for peritoneal metastasis. We present a case of a 14 years old child with peritoneal metastasis from recurrent colorectal cancer.

**Presentation of case:**

Colonoscopy and CT-scan were performed leading to the diagnosis of a stenosing adenocarcinoma of the right colon in 2015. Two pelvic lesions were found at the total body PET scan, suspected for peritoneal metastasis. Neoadjuvant chemotherapy was administered, and restaging CT-scan and magnetic resonance (MRI) highlighted a partial response. The patient underwent right laparoscopic hemicolectomy. The postoperative staging was T4 N1 G3. Seven months after the last cycle of adjuvant chemotherapy, CT-scan revealed two huge abdominal masses. The patient underwent explorative laparotomy and bilateral oophorectomy, positive for metastasis from colorectal cancer and peritoneal washing cytology was positive for neoplastic cells. A CT-scan was performed on December 2017 showed a suspect lesion below the anterior abdominal wall. The case was discussed at the tumour board and the indication for CRS and HIPEC was given. In January 2018 the child underwent complete CRS and HIPEC with no complications. No adjuvant chemotherapy was administered. After 11 months the follow up is negative for the recurrent disease.

**Discussion and conclusion:**

Cytoreduction and HIPEC can be performed even in children as a feasible and safe treatment with successful outcomes. As for adults, an appropriate multidisciplinary pre-operative work up and a correct cases selection is needed to have the best results even regarding the quality of life.

## Highlights

• Cytoreduction (CRS) and HIPEC is a feasible and safe treatment of Peritoneal surface malignancies.
• Multidisciplinary evaluation of the patient is mandatory to obtain positive results.
• Cases of CRS and HIPEC in children are few; it’s important to share every cases with the surgical and oncological community.
• In well selected cases, both disease free survival and overall survival are improved.

## 1 Introduction

Cytoreductive surgery [CRS] combined with hyperthermic peritoneal perfusion with chemotherapy [HIPEC] is nowadays a well-established treatment in patients with peritoneal surface malignancies without extraperitoneal disease [2,3]. Therefore the patients are considered eligible for CRS and HIPEC if they have got a good performance status and don’t have all contraindications for large general surgery. CRS is performed doing a complete resection of all tumoral lesions and invaded organs. Extensive peritonectomy is always performed, and before the administration of HIPEC, all the anastomosis are done. HIPEC is performed by perfusion of high-temperature chemotherapy molecules such as Mitomycin C, Doxorubicin or Cisplatin. The synergy of the two techniques provides long-term disease control, and it is recognised as the first-choice treatment for Pseudomyxoma peritoneii and Peritoneal mesothelioma [4,5]. CRS and HIPEC find very specific indications in well-selected patients with peritoneal carcinomatosis from colorectal, ovarian and gastric cancer in adults.

Among the pediatric population, the use of HIPEC remains debated. Desmoplastic small round cell tumour [DSRCT] has been the first main indication in children and, according to the recent literature, few other tumours have been treated with CRS and HIPEC including colorectal cancer [6]. We present a case of CRS and HIPEC in a 14 years old patient with peritoneal carcinomatosis from adenocarcinoma of the right colon. Only other four pediatric cases of peritoneal metastasis from colon cancer treated by CRS and HIPEC have been described in literature up to now.

## 2 Presentation of case

A 12 years old girl presented to the emergency department in 2015 for hematochezia and anaemia, and she was admitted to the pediatric ward. The patient underwent to colonoscopy with the evidence of an obstructive and ulcerated lesion of the right colon affecting the caecum and the ascending colon. Multiple biopsies were performed, and the histological examination highlighted an infiltrating adenocarcinoma of the right colon, CK20+, CDK 7+. A staging CT-scan of thorax and abdomen confirmed the presence of an 8 cm partially obstructing lesion of the right colon, moreover, two small pelvic nodules were found. The PET-CT scan confirmed the suspect of peritoneal carcinomatosis of the pelvis and the patient underwent two cycles of chemotherapy with FOLFOXIRI. At the re-staging CT-scan and MRI, the primitive tumour showed a partial response to the chemotherapy, while the two pelvic nodules were no more detectable. After discussion with oncologists and paediatricians, the patient underwent laparoscopic right hemicolectomy. At the pathological examination, the diagnosis of signet ring colon carcinoma was confirmed. A tumour presented nerve and blood vessel invasion and 3 out of 57 lymph nodes resected were found positive as secondary localisation. The post-operative staging resulted in T4 N1 M0 G3. The immunohistochemistry showed both KRAS and BRAF wild-type. From December 2016 to April 2017 the patient received eight cycles of FOLFOX.

**Fig. 1:**
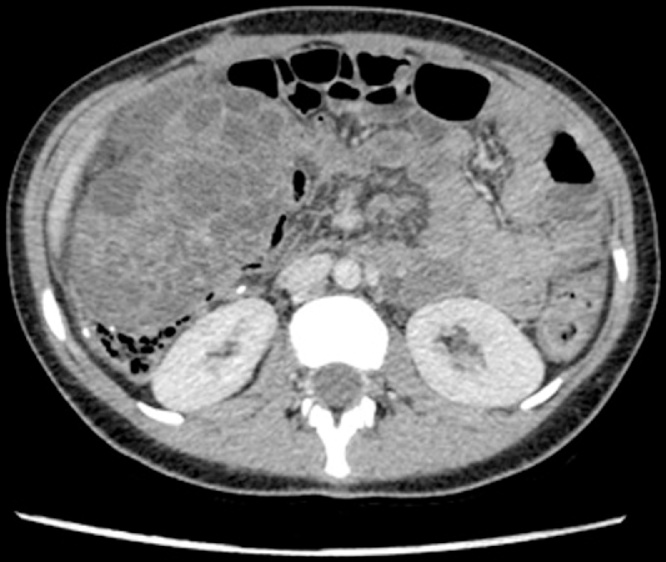
Follow up CT-SCAN of the abdomen that highlight the presence of two bulky pelvic mass both growing from ovaries.

**Fig. 2:**
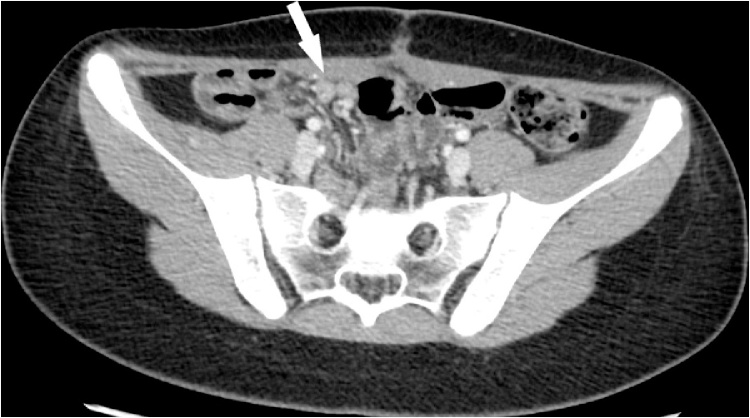
Pre-operative CT-SCAN: peritoneal metastasis were found in the contest of the right rectus muscle and of the mesentery next to the umbilicus’’.

In November 2017 the patient has admitted in the pediatric ward for abdominal pain and a CT-scan of the abdomen highlighted the presence of two bulky pelvic mass both growing from ovaries (Fig. 1). The patient underwent an exploratory laparotomy with bilateral ovariectomy and peritoneal washing during surgery. Both ovaries resulted in the site of metastasis from colon adenocarcinoma at the pathological examination. Nonetheless, the peritoneal washing was positive for neoplastic cells. In December 2017 at the control CT-scan, suspected peritoneal metastasis were found in the contest of the right rectus muscle and of the mesentery next to the umbilicus (Fig. 2). The case was discussed at the tumour board, and the indication for CRS and HIPEC was given.

**Fig. 3:**
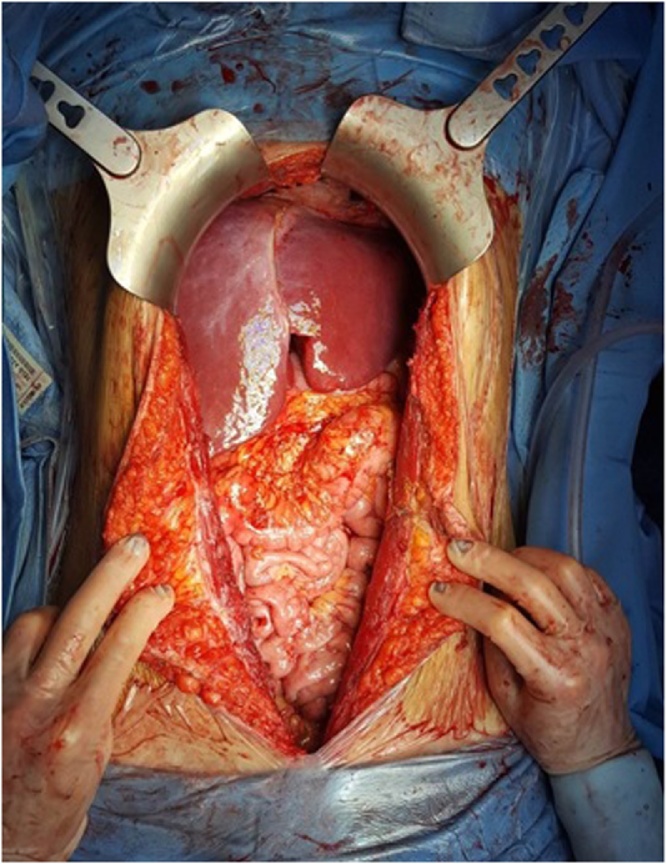
Abdominal cavity after CRS plus HIPEC CCR = 0.

In January 2018 the patient underwent CRS and HIPEC. General anaesthesia was induced with Propofol using the TIVA-TCI Schnider model [effect-site targeting]; also, Methylprednisolone 3 mg/kg was given as part of an internal protocol aimed at reducing cytokine release during major abdominal surgery. Right radial artery was cannulated for invasive blood pressure monitoring, and a central line catheter was inserted into the left internal jugular vein using an US-guided posterior approach. An epidural catheter was placed in lateral decubitus with the median approach, at D12-L1 level. An exploratory laparotomy was performed before to proceed with complete peritonectomy, PCI was 3. The nodule on the anterior abdominal wall was resected. Complete omentectomy and resection of the round ligament were performed, spleen and gallbladder were preserved. At the end of the procedure the CCR was complete (CCR-0) (Fig. 3). One-hour prior HIPEC began, warming tools were stopped [forced-air devices and fluid warmers], and ice packs on the neck, forehead, and armpits were applied. Before to proceed with HIPEC two thoracic drainage were positioned. HIPEC was administrated at 42 °C with 57.6 mg of mitomycin C for 60 min.

The immediate postoperative course was managed in the Intensive Care Unit [ICU], with rapid weaning from mechanical ventilation. No significant variation in creatinine values or renal function was observed. Rapid Lactate clearance occurred. Length of stay in ICU was less than 24 h.

Postoperative analgesia management was complicated by pain secondary to chest drainages not covered by neuraxial technique, so rescue doses of NSAIDs and Morphine were necessary. The peridural catheter was removed during the 7th postoperative day while refeeding occurred on the 6^th^. The patient was discharged on the 14^th^ postoperative day.

The pathological examination showed a complete resection of the tumoral nodules. The case has been discussed with oncologists; no further chemotherapy was indicated. The patient underwent a CT-scan follow-up every three months.

At the last CT-scan, eleven months after the procedure, no evidence of recurrence was shown. The patient is now in good health with a normal quality of life.

## 3 Discussion

The first experience with CRS and HIPEC in children with DSRCT was reported in 2007 by Hayes-Jordan, HIPEC was administrated with cisplatin for 90 min, no postoperative complication occurred. The youngest patient treated by HIPEC reported in the literature was three years old, treated for a peritoneal Ewing sarcoma [7]. Several studies confirm the feasibility of CRS and HIPEC in children, although, between some cohort, high morbidity, up to 66% is reported [8,9]. The major influence on mortality and morbidity can be referred to the histologic type of the tumour, numbers of organs involved and PCI [10]. As for adults, even in children, regardless of the histology, the completeness of cytoreduction is a critical component in the extension of survival. A recent study hypothesised that treating peritoneal malignancies with HIPEC closer from initial diagnosis can decrease morbidity significantly in specific neoplasms of the pediatric population [11]. Hayes-Jordan reported very few complications attributed to HIPEC. What is evident from his series is that severe complications occurred in 28% of the patients as wound infection, small bowel obstruction or urinary tract infection. Wound infections were treated with washing and vacuum therapy, and two patients required to go under surgery to solve bowel obstruction. No complications as anastomotic leakage or bone marrow suppression were observed [12]. In the early phase of his experience Hayes described 3 cases of renal insufficiency secondary to cisplatin. After that, the perioperative hydration protocol was updated, and no more renal failure has been found [13,14]. A recent retrospective analysis reported the rate of complications in a cohort of 2149 patients treated for peritoneal metastasis from a large variety of tumours. The 19.3% of the patient presented a grade III/IV complication. Among these, the 14.3% needed reoperation for fascial dehiscence, hemorrhage, bile leak, and anastomotic leak. Pulmonary embolism showed up in 3.4% of patients. More rarely but gastric perforation, gastric ulcer and cecal perforation occurred [15]. These results are possible when there is a strong collaboration between experienced surgeons, medical oncologists, radiation oncologists, anesthesiologists, radiologists, and ancillary surgical specialists combined with solid nursing, nutrition and physiotherapy infrastructure.

## 4 Conclusions

Selection of the patients and multidisciplinary approach to PSM allow the best results. In pediatric patients, the indications for CRS-HIPEC are very rare, mainly for the small incidence of tumour-types that can take advantage of the treatment. The only practical way to perform prospective randomised clinical trials is to share knowledge creating a large multicenter database as was created by the Peritoneal Surface Oncology International [PSOGI] for appendiceal neoplasms, mesothelioma and rare tumours. Meanwhile, reporting experience even with a small number of patients is essential.

## Conflicts of interest

No conflicts of interest.

## Sources of funding

Non funding were used.

## Ethical approval

Local ethic committee approval n°247/2016 and EudraCT n°2016-000823-24.

## Consent

An informed written consent to use data for clinical research and publication was given at the hospital admission.

The authors confirm that the patient’s parents signed the informed written consent to the surgical procedure and an explicit permission to use personal details and data.

The work was written in line with the SCARE criteria [1].

## Author’s contribution

Sorrentino Lorena, MD: Data collection and author of case report and discussion.

Serra Francesco, MD: Review of surgical technique literature and co-author of discussion.

Cabry Francesca, MD: Review of surgical technique literature and author of introduction.

De Julis Stefano, MD: Consultant of anesthesiology procedure.

Barbieri Elisa, MD: Consultant of anesthesiology procedure.

Girardis Massimo, MD PhD: Consultant of anesthesiology procedure.

Pier Luca Ceccarelli, MD: Consultant of the pediatric clinical case.

Gelmini Roberta, MD PhD: Supervisor and co-author of entire manuscript.

## Registration of research studies

The study is registered at AIFA: EudraCT n°2016-000823-24.

## Disclosure statement

The authors have nothing to disclose.

## Guarantor

Gelmini Roberta, MD PhD.

## Provenance and peer review

Not commissioned externally peer reviewed.
